# Burnout in Infection Control Practitioners During Extended Public Health Crisis Events: A Mixed Methods Systematic Review Protocol

**DOI:** 10.12688/hrbopenres.13915.2

**Published:** 2025-11-21

**Authors:** Sinead Creedon, Anna Trace, Ella Arensman, Maura P Smiddy

**Affiliations:** 1 School of Public Health, University College Cork, Cork, County Cork, T12 XF62, Ireland; 2 School of Applied Psychology, University College Cork, Cork, County Cork, Ireland

**Keywords:** burnout; healthcare; infection control nurse; infection control practitioner; mixed methods systematic review; pandemic.

## Abstract

**Background:**

Infection prevention and control work environments are complex in nature and have long been associated with extended public health crisis events; major global outbreaks, including epidemics, and pandemics. The role of infection control practitioners shifted exponentially during the COVID-19 pandemic and with this rapid change came anxiety, emotional exhaustion and burnout. Burnout at work generally occurs in response to chronic and prolonged exposure to emotionally challenging events, causing emotional exhaustion, feelings of cynicism, and lack of accomplishment at work. This systematic review aims to investigate burnout in infection control practitioners during extended public health crisis events in upper middle and high income countries globally.

**Methods:**

A mixed-methods systematic review will be carried out and will include qualitative, quantitative and primary mixed-methods studies that investigate the elements of burnout and associated factors such as individual factors and organisational challenges, during extended public health crisis events. After an initial scoping literature search, up to six databases will be searched for studies on burnout in relation to infection prevention practitioners. Study quality will be checked using standardised Joanna Briggs Institute critical appraisal tools. The proposed review will follow the Joanna Briggs Institute convergent integrated approach for mixed methods systematic reviews. Following data extraction, quantitative data will be converted into ‘qualitized data’ in the form of textual descriptions.

**Discussion:**

It is well documented that adverse conditions during extended public health crisis events can lead to burnout. This systematic review will investigate burnout in infection control practitioners in upper-middle and high-income countries during public health crisis events. The findings will inform healthcare professionals, senior policy makers and researchers contributing to a richer understanding of burnout and associated factors in infection control practitioners with the aim of informing implementation of support structures in practice.

Protocol Registration Number: PROSPERO 2024 CRD42024508996.

## Introduction

Infection control practitioners (ICPs) have long played pivotal roles in upper middle and high income countries during extended public health crisis events, including the Severe Acute Respiratory Syndrome (SARS) outbreak in 2002, the swine flu pandemic in 2009, and the more recent SARS-CoV-2 pandemic in 2020 ^1^. With each emerging infectious disease event, ICPs have had to manage the heavy burden associated with excessive workloads, working long hours, constantly changing guidelines, managing serious outbreaks, training frontline workers, and implementation of guidelines to help prevent the transmission of infection ^2^. This has contributed significantly to the already high burnout levels in the profession ^3^. The majority of ICPs are trained nurses by profession ^4^ however in some areas, particularly in the United States, this is not the case ^5, 6^. ICPs in the United States hold Certification Board of Infection Control and Epidemiology (CIC ^®^) certification ^7^, which requires them to have completed a post-secondary education in a health-related field with the majority being nurses, but also other healthcare professionals. For this reason, this review will focus on both.

Maslach describes burnout as ‘a prolonged response to chronic emotional and interpersonal stressors on the job’ and is characterised by three core domains: emotional exhaustion, feelings of increased distance from work (cynicism), and reduced professional efficacy ^8^. Burnout is a response to sustained work-related stress, with both organisational factors ^9^ such as the emotional demands of work and workload, individual factors ^8^, such as the person’s ability to cope ^8^, and their personality type ^10^. Burnout is also now recognised by the World Health Organization (WHO) as an occupational phenomenon and defined as “a syndrome conceptualised as resulting from chronic workplace stress that has not been successfully managed” ^11^.

Research strongly suggests that burnout in nurses is prevalent, particularly during the early career period or those who experience redeployment to the front line during extended public health crisis events (e.g., COVID-19) ^12, 13^. Older nurses are also affected, leading to many leaving the profession well before retirement ^14– 17^. Organisational issues, including lack of autonomy ^18^ and poor social supports can also contribute to burnout ^19^, which can result in reduced work efficacy ^20, 21^ and higher rates of absenteeism ^22^. Additionally, patient mortality rates are reported to be higher in hospitals with higher rates of nurse burnout ^23, 24^ and more recently, a study reported a correlation between nurse burnout and the implementation of infection prevention and control measures ^25^. According to the 2022–2023 APIC Mega Survey, around 65% of infection preventionists reported symptoms of burnout, and 70% met criteria for high stress during and after the COVID-19 pandemic. Depression and anxiety rates were 22% and 30%, respectively ^26^. Infection Control Practitioners (ICPs) faced expanded responsibilities and heavier workloads, yet had access to fewer resources. Evidence consistently shows that such adverse conditions during public health crisis events significantly increases the risk of burnout among ICPs ^27^.

## Study aims and review questions

This mixed-methods systematic review (MMSR) aims to evaluate the quantitative, qualitative, and primary mixed-method studies pertaining to infection control practitioner burnout factors (emotional exhaustion; feelings of increased distance from work/cynicism; reduced professional efficacy), and a response to sustained work-related stress, including organisational factors (e.g., the emotional demands of work and workload), individual factors (e.g., a person’s ability to cope), and personality type, during public health crisis events (extended outbreaks, epidemics, and pandemics), in upper-middle to high-income countries as defined by the World Bank country classifications by income level ^28^.

## Methods

### Inclusion criteria

This MMSR protocol, which is visually represented below, incorporates the PICo framework (Population, Phenomenon of Interest, COntext) as recommended by JBI for MMSR ^29^. The PICo framework ( Table 1) will facilitate the identification of the defining characteristics for the study inclusion criteria.

**Table 1.** PICo Framework ^29^.

**Population**	infection control practitioners, including those in dual or job-sharing roles.
**Phenomena ** **of Interest**	Burnout (emotional exhaustion, cynicism and reduced professional efficacy) and other associated factors (organisational) that contribute to the phenomena of interest
**COntext**	Acute and non-acute healthcare settings in upper-middle, and high-income countries globally during extended public health crisis events.

**Population:** The review will consider studies that include only ICPs. Other healthcare staff (including students) will be excluded from the study. Studies will be excluded if they did not differentiate between for example, general nurses and ICPs.

**Phenomena of interest:** This review will consider all studies that investigate the different elements of burnout and other associated factors such as organisational challenges, during public health crisis events as defined above.

**COntext:** This review will consider studies from upper-middle and high-income countries only, and will include ICPs who work in acute and non-acute healthcare settings.

**Types of studies:** This review will consider quantitative (cross-sectional and cohort or longitudinal studies) and qualitative (interviews, focus groups, case studies, image-based methods), and mixed methods studies. Primary mixed-methods studies will be included if the data can explicitly be extracted from the respective components ^30^. Included studies will comprise of published, peer-reviewed, full text articles. Unpublished articles (grey literature) will not be included.

The proposed systematic review will follow the JBI convergent integrated approach for mixed methods systematic reviews ^29^. This methodology is justified as it will allow for a methodical and rigorous approach and provide a contextual understanding of the topic which further enhances reliability by combining different categories of evidence ^31^. This approach is preferable, because the review question can be addressed by both quantitative and qualitative research principles. Following data extraction, quantitative data will then be converted into ‘qualitized data’ in the form of textual descriptions ^32^.

## Search strategy

An initial search of PubMed, Google Scholar, Cochrane, and PROSPERO did not yield any systematic reviews investigating burnout in ICPs hence, this review is the first to examine burnout in this cohort.

The search strategy aims to locate published peer reviewed studies but will exclude peer reviewed abstracts. JBI methodological guidance recommends a three-phase search strategy, the first of which is a broad initial search of at least two databases ^33^. A broad preliminary search of the literature was performed using Google Scholar and OneSearch (UCC Library default search engine) to identify relevant papers. Following the preliminary search, the text words contained in the titles and abstracts of relevant articles, and the index terms used to describe the articles, were used to develop a full search strategy for the following electronic bibliographic databases: MEDLINE (PubMed), CINAHL (EBSCO), Embase (Elsevier), PsycINFO (EBSCO), Scopus (Elsevier), and Web of Science (Clarivate). The search strategy, including keywords and index terms, will be adapted for each included information source. Additionally, reference lists of studies selected for critical appraisal, will be screened for additional studies. Boolean operators and controlled vocabulary terms will be used as required for each database search.

Searches published in all languages will be included. Studies published from November 2002 to August 2024 will be included, in line with the first extended public health crisis event experienced by ICPs in upper-middle and high-income countries; Severe Acute Respiratory Syndrome (SARS), which was first identified in November 2002. A sample search strategy (CINAHL) can be found in Extended Data ^34^

## Study selection

Following the search, all identified citations will be loaded into Mendeley Reference Manager (version 2.117.0/2024) and duplicates removed. These will be imported into the Johanna Briggs Institute (JBI), System for the Unified Management, Assessment, and Review of Information (JBI SUMARI) ^35^ for screening. Following a pilot test, titles and abstracts will then be screened by two independent reviewers for assessment against the inclusion criteria for the review. Potentially relevant studies will be retrieved in full, and their citation details imported into JBI SUMARI ^36^. The full text of selected citations will be assessed against the inclusion criteria by two independent reviewers. Reasons for exclusion of full-text studies that do not meet the inclusion criteria will be recorded and reported in the systematic review. Any disagreements that arise between the reviewers at each stage of the study selection process will be resolved through discussion or with a third reviewer. The results of the search will be reported in full in the final review and presented in a Preferred Reporting Items for Systematic Reviews and Meta-Analyses (PRISMA) flow diagram ^37^.

## Assessment of methodological quality

Quantitative papers (and the quantitative component of mixed methods papers) selected for retrieval will be assessed by two independent reviewers for methodological validity prior to inclusion in the review using standardized critical appraisal instruments from JBI SUMARI ^38^.

Qualitative papers (and qualitative component of mixed methods papers) selected for retrieval will be assessed by two independent reviewers for methodological validity prior to inclusion in the review using the standardized critical appraisal instrument from JBI SUMARI ^38^.

Authors of papers will be contacted to request missing or additional data for clarification, where required. Any disagreements that arise between the reviewers will be resolved through discussion or with a third reviewer. The results of critical appraisal will be reported in narrative format and by visual representation in a table.

Following critical appraisal, studies that do not meet the required quality threshold will be excluded.

## Data extraction

Quantitative and qualitative data will be extracted from studies included in the review by two independent reviewers using the standardised JBI data extraction tool for MMSR following a convergent integrated approach, in JBI SUMARI ^35^ or in RevMan (version 5.0). The data extracted will include specific details about the populations, study methods, phenomena of interest, context etc., and outcomes of relevance to the review question(s). Specifically, quantitative data will be composed of data-based outcomes of descriptive and/or inferential statistical tests ^32^. In addition, qualitative data will be composed of verbatim themes or subthemes with corresponding illustrations and will be assigned a level of credibility depending on the quality of the data ^32^. This protocol was developed in line with the PRISMA-P checklist ^39^, which can be found in Extended Data ^34^.

## Data transformation

The quantitative data will be converted into “qualitized” data after data extraction. This will involve transformation into textual descriptions or narrative interpretation of the quantitative results in a way that answers the review questions ^29^.

## Data synthesis and integration

This will involve assembling the qualitized quantitative data with the qualitative data. Assembled data will be categorised and pooled together based on similarity in meaning, to produce a set of integrated findings.

## Dissemination

This study will be submitted for publication in scientific journals and will be publicly available as an open access paper.

## Study status

The study has been registered on PROSPERO (CRD42024508996). We are currently undergoing systematic searches. Screening, data extraction, synthesis and integration will take place thereafter.

## Discussion

Although infection prevention and control is a relatively young discipline, it is well established that adverse conditions during public health crisis events can lead to burnout in ICPs ^3^, who are experiencing serious mental health challenges, particularly now in the aftermath of the COVID-19 pandemic ^2^. A call to action is urgently required to address burnout in ICPs during extended public health crisis events. It is important that policymakers, healthcare leaders and researchers prioritise strategies and interventions to mitigate burnout in ICPs. This could include integrating burnout prevention strategies into infection prevention and control policies and frameworks; embedding mental health support into organisational frameworks, implementing surge staffing policies during extended public health crisis events, and ensuring ICPs have access to resilience training, supports, and adequate resources. IPC wellbeing is put at risk if not addressed, but also the standard of IPC programs and patient safety. Focused and sustained action will be essential to maintaining a resilient workforce that can effectively address future public health emergencies.

## Ethics and consent

Ethical approval and consent were not required.

## Data Availability

No data are associated with this article. Zenodo: Preferred Reporting Items for Systematic Review and Meta-Analysis Protocols (PRISMA-P) checklist for “Burnout in Infection Control Practitioners during public health crisis events: A Mixed Methods Systematic Review Protocol” ( https://zenodo.org/records/17580826) ^25^ Zenodo: CINAHL (EBSCO) Sample Search Strategy ( https://zenodo.org/records/13961043) ^25^ This work © is licensed under Creative Commons Attribution 4.0 International. To view a copy of this license, visit https://creativecommons.org/licenses/by/4.0/
