# Supplementary material for: Burnout in Infection Control Practitioners During Extended Public Health Crisis Events: A Mixed Methods Systematic Review Protocol
Source: HRB Open Res. 2025 Nov 21;7:69. Originally published 2024 Oct 29. [Version 2] doi: 10.12688/hrbopenres.13915.2 (PMC12586984; doi:10.12688/hrbopenres.13915.2)
Supplement: Peer review history

# Reviewer report: Reviewer response for version 2

**DOI:** 10.21956/hrbopenres.15732.r51705

**Published:** 2025-12-11

**Authors:** Brenna Doran

**Affiliations:** 1 University of California San Francisco, San Francisco, Canada

The revisions made to this manuscript have substantially elevated the study's methodological rigor and clarity. By explicitly defining 'extended public health crisis events' and justifying the exclusion of Ebola based on income levels, the authors have established a precise and logical scope. The decision to expand the search strategy beyond English-language results, coupled with the use of World Bank classifications, reduces potential selection bias and enhances the study's external validity. Furthermore, the refinement of the PICO framework and inclusion criteria ensures the review is reproducible and robust. The integration of specific burnout statistics in the introduction provides necessary context and a stronger rationale for the research. Additionally, the new 'call to action' in the discussion successfully translates the findings into practical implications for stakeholders. The comprehensive update of references ensures the work is grounded in the most current literature. Collectively, these amendments result in a scientifically sound and impactful contribution to the understanding of healthcare worker burnout.

Is the study design appropriate for the research question?

Yes

Is the rationale for, and objectives of, the study clearly described?

Yes

Are sufficient details of the methods provided to allow replication by others?

Partly

Are the datasets clearly presented in a useable and accessible format?

Yes

Reviewer Expertise:

USA Infection Prevention and control staffing, retention/ attrition, mental health, and burnout

I confirm that I have read this submission and believe that I have an appropriate level of expertise to confirm that it is of an acceptable scientific standard.

# Reviewer report: Reviewer response for version 2

**DOI:** 10.21956/hrbopenres.15732.r51704

**Published:** 2025-11-26

**Authors:** Jane Chudleigh

**Affiliations:** 1 King's College London, London, England, UK

Thank you for responding to the query raised previously. I agree with the revised suggested wording i.e.,:

*Context: This review will consider studies from upper-middle and high-income countries and will include ICPs who work in both acute hospital settings and non-acute settings such as the community and long-term care.*

 Please could this be applied to the appropriate place in the article. 

 Thanks

 Jane

Is the study design appropriate for the research question?

Yes

Is the rationale for, and objectives of, the study clearly described?

Yes

Are sufficient details of the methods provided to allow replication by others?

Yes

Are the datasets clearly presented in a useable and accessible format?

Not applicable

Reviewer Expertise:

Infection control, genetics

I confirm that I have read this submission and believe that I have an appropriate level of expertise to confirm that it is of an acceptable scientific standard.

# Reviewer report: Reviewer response for version 1

**DOI:** 10.21956/hrbopenres.15263.r50986

**Published:** 2025-11-04

**Authors:** Emma Burnett

**Affiliations:** 1 Fatima College Of Health Science, Abu Dhabi, United Arab Emirates

Thank you for submitting this manuscript. It is a well-conceived and methodologically rigorous mixed-methods systematic review that addresses a highly relevant and underexplored topic: burnout among Infection Control Practitioners (ICPs) during public health crises. I enjoyed reading it and have highlighted some aspects which could further enhance this paper:

 Research aims are clear and justified, but the rationale could be broadened by explicitly linking the study to global infection control practice, including under-represented regions. Also consider linkage to other major IPC crises (Ebola, MERS, Avian Influenza)

 Perhaps consider defining explicitly what constitutes a “public health crisis event” and whether studies addressing ongoing but non-pandemic stressors (e.g. AMR outbreaks) will be excluded.

 The three-phase JBI search process is clearly described, but excluding non-English papers risks omitting relevant international literature, especially from Asia, Europe, and South America. Given the AI-assisted translation tools that are now freely available, you may wish to consider including these studies to strengthen global representation.

 The discussion section could also be further strengthened by considering

 1. What specific insights or policy outcomes are expected.

 2. How these findings might influence workforce well-being strategies or organizational interventions for ICPs.

 3. Is there a short, impactful “call to action” you could add for policymakers and researchers.

Is the study design appropriate for the research question?

Yes

Is the rationale for, and objectives of, the study clearly described?

Partly

Are sufficient details of the methods provided to allow replication by others?

Yes

Are the datasets clearly presented in a useable and accessible format?

Yes

Reviewer Expertise:

Infection Prevention and Control

I confirm that I have read this submission and believe that I have an appropriate level of expertise to confirm that it is of an acceptable scientific standard.

# Reviewer report: Reviewer response for version 1

**DOI:** 10.21956/hrbopenres.15263.r50987

**Published:** 2025-10-31

**Authors:** Brenna Doran

**Affiliations:** 1 University of California San Francisco, San Francisco, Canada

**Peer Reviewer Comments**

**Overall Assessment**

 This is an exceptionally timely and critical study protocol that addresses an urgent issue in global healthcare: burnout in Infection Control Practitioners (ICPs) during public health crises. The authors have articulated a clear rationale, and the decision to use a mixed-methods systematic review (MMSR) protocol following the rigorous JBI convergent integrated approach is highly appropriate for synthesizing both quantitative and qualitative evidence to gain a contextual understanding of this complex topic. The detailed methodology, including the PICO framework and the thorough, three-phase search strategy, demonstrates a strong foundation for what promises to be the first systematic review of burnout in this crucial cohort , ensuring the findings will be robust and invaluable for policy makers, researchers, and healthcare professionals globally.

**Review comments**

**Introduction**

 1. Introductory Crisis Events: Strengthen the opening sentence by including additional public health crises (e.g., avian flu, Ebola). A citation is required for this statement unless it is generalized. Citation for consideration:

- refer to 1,2

2. 2 ^nd^ sentence - supporting Workload Statements: The statement regarding excessive workloads and working long hours must be supported with references. Please see the citation suggestions under comment #3.

 3. 3 ^rd^ sentence, include additional international citations to support the statement on “high burnout levels”. The current suggestions are excellent but are US-based; adding international sources will support the global lens. A  few to consider (below are US based, recommend adding additional international citations)

- refer to 3,4,5,6,7

4. Sentence 4 – need citations to support statement (1) that majority of  ICPs are nurses; (2) in US are not, and (3) CIC requires a post-secondary degree

- (1) Majority of IPC are RN
  - refer to 8
- (2) US IPC Demographics
  - refer to 9,10
- (3) CIC requirement : https://www.cbic.org/CBIC/Candidate-Handbook/Eligibility-Guidelines.htm

Introduction 3 ^rd^ paragraph (below comment applies to paragraph as a whole)

 5. Recommend framing burnout in nursing first, include international citations (to help support your global lens) then talk about early/ redeployment and older nurses. Add data (such as percentage of RNs leaving the profession after COVID to strengthen these statements and add citations (just a few to consider),

- refer to 11,12,13

6. This statement/ section can be strengthened. “Additionally, patient mortality rates are reported to be higher in hospitals with **higher rates of nurse burnout** and more recently, a study reported **a correlation between nurse burnout and the implementation of infection prevention and control measure**s.”  From your perspective, what is the central problem you are hoping this research will prevent?  IPC Attrition, retention, patient harm?  Also, need to add specific data points and citations to this statement.

 7. **Population**: Recommend being clearer in inclusion/ exclusion criteria.  Examples, are you looking at IPCs who work in just healthcare organizations only? Are you including Public Health and industry?  Does the IPC need to only work in IPC? What about IPCs who have dual roles? Are you going to include data for IPC who are currently employed as IPCs or former IPCs too? Finally, consider how you will approach if paper is on HCW more generally and IPCs are a subset of the data reported.

 8. **Phenomena of interest** – How will your inclusion criteria define “burnout” Earlier the MBI and WHO definitions were used.  Are you including all papers on burnout or just those with a burnout inventory/ assessment included?

 9. **Context:** How are you defining upper-middle and high-income countries? Are you using an existing definition, if so, list what it is.  If not, how are you defining what meets these criteria?

 10. **Time-frame:** Recommend adding and/ or defining the time frame for your review.  Are you reviewing only papers with research studies that occurred or centered during COVID (need to include how you are defining that time frame) or are you also looking at before and/or after COVID? 

 11. **Search Strategy** – as you are conducting a global review, consider accepting papers written in other languages as there is AI and translation options available.  

 12. “Studies published from November 2002 to August 2024 will be included, as the first public health crisis event experienced by ICPs was Severe Acute Respiratory Syndrome (SARS), first identified in November 2002.” Recommend included as much of 2025 as there are some manuscripts that have been and will be published this year that will add to your review. Also, for consideration update from date published to date of research study performed (as there have been a couple of papers published in 2025 from data from COVID).

 13. **Study selection** – consider accepting adding inclusion criteria for major or minor research articles and excluding abstracts/ commentaries as all may show up during your systemic review.

 14. **Discussion** – overall opportunity to add additional research papers to strengthen and add international lens. See comments 1,3-5.

 “Infection  prevention and control (IPC) is a relatively young discipline dating back to the 1970s.” Statement need citation.  It is also at slight odds with **Introduction; first sentence** that IPC “public health crisis events” in 2002.  The two different statements need to be addressed to align with clear lens and scope of the paper.

 “The findings will inform **healthcare professionals,** senior policy makers and researchers  and will help contribute to a richer understanding of  burnout and associated factors in infection control practitioners.” This closing would benefit from a stronger more impactful closing.  What is the goal of this review? What is the call to action? What do you envision as next steps? What is the role of future research? 

 What is your elevator pitch?

 “…this review provides an urgent call to action for healthcare leaders and policy makers to implement targeted, evidence-based strategies to improve working conditions and protect the well-being of this critical workforce….and simultaneously set the agenda for future research aimed at developing and validating effective, scalable solutions to mitigate infection control practitioner burnout."

Is the study design appropriate for the research question?

Yes

Is the rationale for, and objectives of, the study clearly described?

Yes

Are sufficient details of the methods provided to allow replication by others?

Partly

Are the datasets clearly presented in a useable and accessible format?

Yes

Reviewer Expertise:

USA Infection Prevention and control staffing, retention/ attrition, mental health, and burnout

I confirm that I have read this submission and believe that I have an appropriate level of expertise to confirm that it is of an acceptable scientific standard, however I have significant reservations, as outlined above.

## References

[1] Park, S.Y., Cheong, H.S., Kwon, K.T., Sohn, K.M., Heo, S.T., Lee, S., Chung, U.S. and Lee, S.H., 2023. Guidelines for infection control and burnout prevention in healthcare workers responding to COVID-19. Infection & Chemotherapy, 55(1), p.150. 10.3947/ic.2022.0164PMC1007943837021430

[2] Torriani, F. and Taplitz, R., 2012. History of infection prevention and control. Infectious Diseases, p.76.

[3] : Associations among infection prevention professionals' mental/physical health, lifestyle behaviors, shift length, race, and workplace wellness support during COVID-19. *American Journal of Infection Control* .2023;51(1) : 10.1016/j.ajic.2022.04.004 62-69 10.1016/j.ajic.2022.04.004 PMC927914135843751

[4] : Impacts of the COVID-19 pandemic on the infection prevention and control field: Findings from focus groups conducted with association for professionals in infection control & epidemiology (APIC) members in fall 2021. *American Journal of Infection Control* .2023;51(9) : 10.1016/j.ajic.2023.02.013 968-974 10.1016/j.ajic.2023.02.013 PMC998553536882121

[5] : Impact of the COVID-19 pandemic on infection control nurses: A path analysis of job stress, burnout, and turnover intention. *American Journal of Infection Control* .2024;52(6) : 10.1016/j.ajic.2024.01.001 683-687 10.1016/j.ajic.2024.01.001 38218329

[6] : The role of spirituality, religiosity, and self-care on infection preventionist well-being: Results from a national survey in the United States. *American Journal of Infection Control* .2024;52(6) : 10.1016/j.ajic.2023.12.006 726-730 10.1016/j.ajic.2023.12.006 PMC1308267538122935

[7] : The influence of hospital leadership support on burnout, psychological safety, and safety climate for US infection preventionists during the coronavirus disease 2019 (COVID-19) pandemic. *Infection Control & Hospital Epidemiology* .2024;45(3) : 10.1017/ice.2023.184 310-315 10.1017/ice.2023.184 PMC1093349837702064

[8] Reese, S.M., Shackelford, A., Knepper, B., Merrill, K., Nicholson, F., Baghagho, E., Doran, B., Stoltzfus, H. and Crapanzano-Sigafoos, R., 2025. Retaining your Infection Preventionist: Top ten factors that lead to retention in the US. American Journal of Infection Control. 10.1016/j.ajic.2025.06.01240541666

[9] Merrill, K., Cervantes, D., Hebden, J.N., Pogorzelska-Maziarz, M., Piatek, D., Monsees, E. and Hessels, A., 2024. Infection preventionists in public health, consultant and academic roles: Results from the 2020 APIC MegaSurvey. American journal of infection control, 52(3), pp.261-266. 10.1016/j.ajic.2023.09.00137689123

[10] Pogorzelska-Maziarz, M., Monsees, E. and Hessels, A., 2023. APIC Megasurvey 2020: methodology and overview of results. American journal of infection control, 51(3), pp.241-247. 10.1016/j.ajic.2022.12.00236535317

[11] Rizzo, A., Yıldırım, M., Öztekin, G.G., Carlo, A.D., Nucera, G., Szarpak, Ł., Zaffina, S. and Chirico, F., 2023. Nurse burnout before and during the COVID-19 pandemic: a systematic comparative review. Frontiers in Public Health, 11, p.1225431. 10.3389/fpubh.2023.1225431PMC1050788237732086

[12] Zareei, M., Tabanejad, Z., Oskouie, F., Ebadi, A. and Mesri, M., 2022. Job burnout among nurses during COVID-19 pandemic: A systematic review. Journal of education and health promotion, 11, p.107. 10.4103/jehp.jehp_797_21PMC909365235573618

[13] Galanis, P., Vraka, I., Fragkou, D., Bilali, A. and Kaitelidou, D., 2021. Nurses' burnout and associated risk factors during the COVID‐19 pandemic: A systematic review and meta‐analysis. Journal of advanced nursing, 77(8), pp.3286-3302. 10.1111/jan.14839PMC825061833764561

# Reviewer report: Reviewer response for version 1

**DOI:** 10.21956/hrbopenres.15263.r43211

**Published:** 2024-12-17

**Authors:** Jane Chudleigh

**Affiliations:** 1 King's College London, London, England, UK

The authors propose a systematic review of 'Burnout in Infection Control Practitioners During Public Health Crisis Events'. This is both timely and important given events in recent years. 

 The background/rationale is articulated well.

 The methods are appropriate and described in detail. Please clarify if you intend to include papers from the community, primary care setting or is this just the hospital setting?

 Please could the authors simply provide clarification about the setting as indicated in the comments  i.e., hospital, community and/or primary care. This is my only reservation but there were no other options to choose from that seemed to fit.

Is the study design appropriate for the research question?

Yes

Is the rationale for, and objectives of, the study clearly described?

Yes

Are sufficient details of the methods provided to allow replication by others?

Yes

Are the datasets clearly presented in a useable and accessible format?

Not applicable

Reviewer Expertise:

Infection control, genetics

I confirm that I have read this submission and believe that I have an appropriate level of expertise to confirm that it is of an acceptable scientific standard, however I have significant reservations, as outlined above.

# Response

**Published:** 2025-01-06

**Authors:** Sinead Creedon

**Affiliations:** School of Public Health, University College Cork, Cork, Ireland

Dear Jane,

 Many thanks for taking the time to review my paper.

 In the context part of my PICO, I have outlined ICPs who have worked in both acute and non-acute settings, which leaves it broad enough to include all types of healthcare settings. Further, most of the papers for this review are from the US and the word primary care can have a different meaning in different countries, so I think it might be best to keep it broad and use the term community, which would cover primary care. Would this be a more appropriate entry for the Context element of PICO?:-

*Context: This review will consider studies from upper-middle and high-income countries and will include ICPs who work in both acute hospital settings and non-acute settings such as the community and long-term care.*

 Thank you,

 Sinead
